# Psychological Interventions Targeting Maternal Role Development and Identity in Perinatal Mental Health: A Systematic Review with Qualitative Synthesis

**DOI:** 10.3390/healthcare14131958

**Published:** 2026-07-02

**Authors:** Lorena Gutiérrez Hermoso, Cecilia Peñacoba Puente, Carmen Écija Gallardo, Livia Gomes Viana Meireles, Patricia Catalá Mesón

**Affiliations:** 1 Department of Psychology, Faculty Health of Sciences, Rey Juan Carlos University, 28932 Madrid, Spain; lorena.gutierrezh@urjc.es (L.G.H.); cecilia.penacoba@urjc.es (C.P.P.); carmen.ecija@urjc.es (C.É.G.); 2 Institute of Physical Education and Sport, Federal University of Ceara, Fortaleza 6021, Brazil; liviagviana@ufc.br

**Keywords:** maternal role acquisition, maternal identity transition, perinatal mental health, psychological support programs

## Abstract

**Highlights:**

**What are the main findings?**

- Psychological and psychoeducational programs supporting maternal role acquisition during pregnancy and postpartum improve maternal role adjustment, competence, and self-efficacy.
- Interventions focused on maternal role acquisition are associated with reductions in postpartum depressive symptoms and perceived stress, even when mental health outcomes are not the primary target.

**What are the implications of the main findings?**

- Supporting maternal role acquisition may represent a promising preventive strategy within perinatal mental health care.
- Integrating individualized and culturally sensitive psychological accompaniment into routine perinatal services could enhance maternal, infant, and family health outcomes.

**Abstract:**

**Background**: Maternal identity is the perception and recognition of a woman as a mother. Within this emerging identity, the maternal role takes on special importance as a manifestation of the set of responsibilities that a woman assumes in the care and upbringing of her baby. Respectful professional accompaniment during the period of maternal role acquisition is key to perinatal mental health and secure bonding with the baby. The main objective of this systematic review with narrative synthesis was to analyze the effects of psychological support programs aimed at maternal role acquisition during the transition to motherhood. **Methods**: Studies with experimental and quasi-experimental designs addressing maternal role acquisition in pregnant or postpartum women were included. A systematic search was conducted in PsycINFO, MEDLINE, PubMed and SCOPUS from inception to March 2025 following PRISMA recommendations. Due to the heterogeneity in study designs, interventions and outcome measures, a narrative synthesis was performed instead of a meta-analysis. **Results**: A total of 11 studies were extracted with a total sample of 1244 women, including five randomized controlled trials and six quasi-experimental studies. Psychological support programs focusing on maternal role acquisition generally showed improvements in maternal identity construction, self-efficacy and maternal competence, although not all findings reached statistical significance. In addition, several studies reported reductions in postnatal depressive symptoms, as well as improvements in subjective well-being and maternal role perception. **Conclusions**: results suggest that psychological support programs targeting maternal role acquisition may represent a promising approach for supporting perinatal mental health. However, the evidence should be interpreted with caution due to methodological limitations and heterogeneity across studies. In fact, most included studies were conducted in Eastern cultural contexts (Iran, China), limiting generalizability to Western populations without further adaptation and validation. Additionally, incomplete reporting of standardized effect sizes and precision measures across studies limits the quantitative interpretation of the findings. This review was not prospectively registered, and title/abstract screening was conducted by a single reviewer, increasing the risk of selection bias. Further research using rigorous and standardized designs is needed to clarify the effectiveness and generalizability of these interventions.

## 1. Introduction

Many women globally decide to become mothers. Socially, motherhood is idyllically drawn as a desired experience to achieve a high degree of well-being. However, rapid political, economic and social changes influence women’s psychological development during this experience, affecting their ability to cope with motherhood [1].

The evolution of maternal identity is crucial in psychological development. Traditionally, maternal identity is defined as the perceptions and emotional states women express about their relationship with their babies after childbirth. This ongoing adaptation process involves adopting new behaviors, accompanied by various emotional, physical, and relational changes. This journey, known as the maternal role, is one of the primary role women assume in motherhood, providing the foundation for mental health and neonatal development [2].

In this context, it is important to distinguish between related but conceptually distinct constructs. Maternal identity transition refers to the psychological and developmental process through which a woman integrates the identity of becoming a mother. Maternal role acquisition is defined as the dynamic process through which women develop the skills, behaviors, and confidence required to perform maternal functions. In contrast, maternal role attainment refers to the degree to which a woman achieves competence, satisfaction, and integration of the maternal role in her identity, representing a more consolidated outcome of this process [3].

Acquisition of the maternal role is defined as the dynamic process through which women develop the skills, behaviors, confidence, and identity required to adapt to motherhood and effectively care for their infant. In this context, maternal role attainment is understood as the degree to which a woman achieves competence, confidence, and satisfaction in performing this role. It significantly benefits women’s parenting and well-being, favoring the transition to motherhood [4,5]. However, previous research points to loss of emotional control and feelings of inadequacy of the maternal role as demands unmet by health care services [6]. Epidemiological studies, conducted after the COVID-19 pandemic, show an increase in emotional problems such as anxiety and depression during gestation in Hispanic women, and an increase in the consumption of anxiolytics during postpartum [7]. In the United States, 30% of pregnant and postpartum women report an increase in anxious symptoms, and 20% report depressive problems, figures that are expected to increase in the coming years, endangering the adaptive integration of the maternal role during motherhood [8].

One of the explanations that support these desolate results lies in the understanding of the period that encompasses the integration of the maternal role. According to the new contributions of Meighan & Mercer [9], the process of maternal role acquisition involves the performance and competence to perform maternal behaviors in accordance with that role. This process would begin in pregnancy, characterized by waiting to prepare for the baby’s arrival; it would continue during childbirth, where the mother’s bond and attachment to the baby is strengthened; this process would end in the postpartum period, where the objective is to strengthen confidence and satisfaction with the care provided to the baby.

In line with the contributions of Mercer & Ferketich [2], professional support should address four phases directed towards the acquisition of the maternal role in her identity: anticipatory, formal, informal and personal stages. These four phases overlap with each other and are reflected in the mother–baby dyad, in the mother’s performance with her social circle and in coping with stressful situations [10,11].

From a public health perspective, the acquisition of the maternal role constitutes a key determinant of maternal and child health outcomes. Difficulties in the integration of this role have been associated not only with impaired maternal mental health, but also with suboptimal mother–infant interaction, reduced adherence to health recommendations, and increased use of health care services. Therefore, supporting women during the transition to motherhood should be considered a priority preventive strategy within perinatal care, with potential long-term benefits for families and health systems.

Despite governmental efforts to improve maternal and child health, a recent systematic review questions the impact of accompaniment programs on maternal competence and well-being related to women’s identity [11,12,13,14]. However, the past decade has seen significant progress in implementing psychological programs for perinatal mental health. Multiple clinical trials have demonstrated improvements in depressive symptoms among pregnant women and mothers [15].

Nevertheless, existing interventions differ substantially in their theoretical foundations, timing (antenatal versus postnatal), content, duration, and modes of delivery. Moreover, many programs primarily focus on the reduction in psychological symptoms, such as anxiety or depression, without explicitly addressing the acquisition of the maternal role as a core developmental process. This heterogeneity limits comparability across studies and complicates the identification of key intervention components that effectively support maternal identity and competence.

This progress may explain the current increase in studies focusing on interventions addressing mental health problems in expectant mothers. It is essential to promote programs that focus on supporting motherhood, as this emerging identity acts as a protective factor against perinatal mental health problems. Framing motherhood as a developmental transition rather than solely as a period of psychological risk enables the design of preventive interventions that can be integrated into routine perinatal care. Therefore, the aim of this systematic review with qualitative synthesis is to analyze the content and effects of psychological intervention or support programs focused on the acquisition of the maternal role during the transition to motherhood.

## 2. Materials and Methods

This systematic review was conducted according to the Preferred Reporting Items for Systematic Reviews and Meta-Analyses (PRISMA) checklist [16,17]. The PRISMA checklist is included as Supplementary Materials (Table S1). The review protocol was not prospectively registered.

Search strategy was carried out using the terms “maternal role transition” OR “transition to motherhood” OR “maternal role competence” AND “psychoeducation” OR “psychological program” OR “psychological intervention” OR “psychological therapy”. Combinations of these terms were searched for title, abstract, and keywords. The search strategy was adapted for each database (APA PsycInfo, MEDLINE, PubMed and SCOPUS) using a combination of free-text terms and controlled vocabulary, included Medical Subject Headings (MeSH) in PubMed and Thesaurus terms in PsycINFO. Boolean operators (“AND”, “OR”) were applied to combine the search terms, and database-specific field tags and truncation were used where appropriate. The full search strategies for all databases, including search strings, filters, and dates of the last search, are provided in Supplementary Table S2 in accordance with PRISMA 2020 recommendations.

Searches were conducted in the title, abstract and keywords fields. Language restrictions (English and Spanish) were applied during the screening phase. No study design filters were applied during the search stage.

No restrictions regarding year of publication were applied. The reference lists of all eligible records were reviewed to identify additional studies that met the inclusion criteria. Grey literature was not included.

The final search for all databases was conducted in March 2025. A detailed example of the full search strategy for all databases is provided in Supplementary Table to ensure reproducibility.

### 2.1. Eligibility Criteria

PICO framework was used and inclusion criteria were as follows: (1) studies with pregnant or postpartum women; (2) studies containing assessment measures on components related to maternal role or maternal identity transition and/or acquisition; (3) studies including intervention programs or psychological accompaniment focused on maternal role or maternal identity integration; (4) intervention program protocols, randomized controlled trials, quasi-experimental studies and/or observational studies with comparison groups targeting maternal role acquisition; (5) studies published in English and Spanish.

Exclusion criteria were those studies that met the following characteristics: (1) participating women with children older than 2 years of age; (2) studies that did not contain any type of evaluation measure on maternal role acquisition or on the transition to motherhood to evaluate the effectiveness of the programs; (3) case studies, qualitative studies without comparison groups, and purely descriptive studies; (4) program protocols that did not include work on any of the components that favor the transition to motherhood or the maternal role; (5) systematic reviews or meta-analyses of topics other than the efficacy of perinatal maternal role accompaniment programs; (6) studies published in languages other than English or Spanish.

### 2.2. Study Selection Process

After applying the search strategy, the study selection process was conducted in two phases. First, titles and abstracts were screened independently by one reviewer (LGH) to identify potentially eligible studies, with subsequent supervision and verification by, two additional reviewers (PCC and CPP).

Full-text versions of the selected records were then assessed for eligibility according to the predefined inclusion and exclusion criteria.

Any discrepancies during the selection process were resolved through discussion and consensus, with the involvement of a fourth reviewer (CEG) when necessary.

### 2.3. Data Extraction

The elements extracted from each article included in the review included the following details: reference, year of publication, geographic location with respect to the country where the study was carried out, characteristics of the participants, details of the intervention (application modality, number of sessions, design according to the CONSORT guide, program objectives, variables and evaluation instruments) and results. Data extraction was performed using a standardized extraction form to ensure consistency across studies.

Due to substantial heterogeneity in study designs, intervention characteristics, outcome measures, and follow-up periods, a meta-analysis was not considered appropriate, and a narrative synthesis approach was adopted.

### 2.4. Risk of Bias and Methodological Quality Assessment

The risk of bias was evaluated using the Cochrane Collaboration Risk of Bias tool (RoB 2.0) [18]. This tool was applied exclusively to randomized controlled trials included in the review. Non-randomized studies were assessed using the ROBINS-I tool (Risk Of Bias In Non-randomized Studies of Interventions).

Three reviewers independently assessed the methodological quality of the included records (LGH, PCC, and CPP). Disagreements were resolved through discussion, and when consensus was not reached, a fourth reviewer (CEG) was consulted.

**Figure 1:**
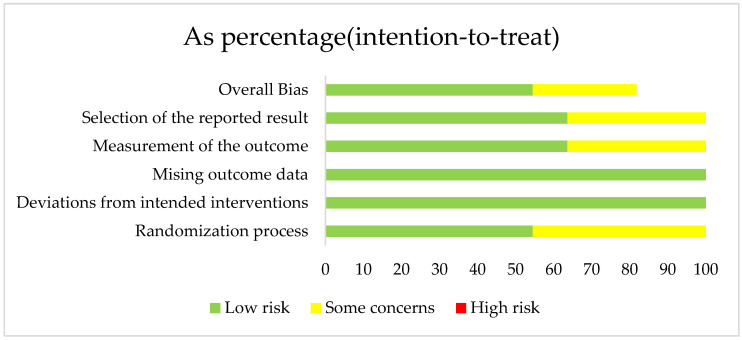
Risk of bias summary for randomized and non-randomized studies. Each domain is rated as low risk (green), some concerns/moderate risk (yellow), or high risk (red), according to RoB 2.0 and ROBINS-I criteria. Domains include: bias arising from the randomization process, deviations from intended interventions, missing outcome data, measurement of the outcome, and selection of the reported result (for randomized studies), as well as confounding, selection of participants, classification of interventions, deviations from intended interventions, missing data, measurement of outcomes, and selection of reported results (for non-randomized studies).

**Figure 2:**
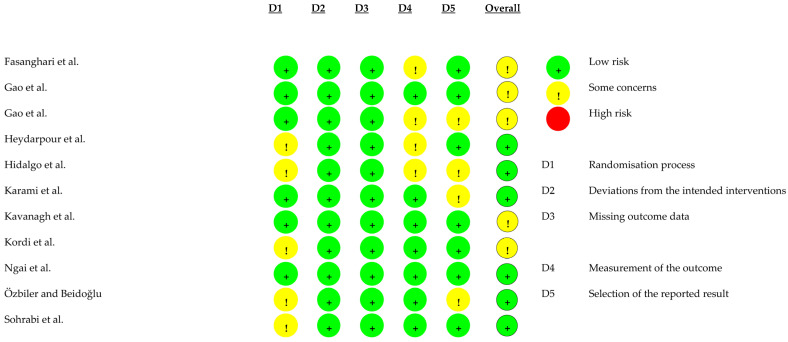
Risk-of-bias graph showing the proportion of studies classified as low risk, some concerns/moderate risk, or high risk across each domain, according to RoB 2.0 and ROBINS-I criteria [19,20,21,22,23,24,25,26,27,28,29].

Judgments for each study were made following the RoB 2.0 and ROBINS-I domains: bias arising from the randomization process, bias due to deviations from intended interventions, bias due to missing outcome data, bias in measurement of the outcome, and bias in selection of the reported result. Each domain was rated as “low risk,” “some concerns,” or “high risk of bias,” leading to an overall risk of bias judgment for each study. Figure 1 and Figure 2 present the risk of bias assessments of the included randomized controlled trials. The results of the risk-of-bias assessment for each study are presented in Supplementary Table S3 to ensure transparency at the study level.

### 2.5. Certainty of Evidence Assessment

The certainty of evidence for the main outcomes was evaluated using the GRADE (Grading of Recommendations Assessment, Development and Evaluation) approach. Two critical outcomes were assessed: (1) maternal role acquisition and (2) maternal self-efficacy/competence.

The GRADE assessment considered the following domains: risk of bias, inconsistency, indirectness, imprecision, and publication bias. Given the inclusion of both randomized and non-randomized studies, and the predominance of quasi-experimental designs, the initial certainty rating was determined accordingly and subsequently downgraded based on the presence of methodological limitations.

Summary of Findings (SoF) tables were developed to present the overall certainty of the evidence and are provided in the Supplementary Materials (Table S4).

## 3. Results

The included studies showed substantial variability in design, intervention characteristics, and outcome measures, which limited comparability across studies. Based on the search strategy, 1215 records were identified through database searching (PsycINFO, MEDLINE, PubMed and SCOPUS), along with 32 additional articles identified through manual searches of reference lists. All search strategies were combined at the identification stage, following PRISMA 2020 guidelines, and duplicate records were removed prior to screening. After removing duplicates and excluding records based on language (English or Spanish) and availability of full text, 852 records were retained for title and abstract screening. Of these, a total of 730 records were excluded for not meeting the inclusion criteria.

Subsequently, 122 full-text articles were assessed for eligibility. However, 89 were excluded for the following reasons: 15 were inaccessible, 46 were not relevant to the variables addressed, 14 had inadequate designs for the objectives of the review, and 14 were intervention protocols focused exclusively on reducing postpartum depressive symptoms. Of the 33 records selected, 22 were excluded: 2 due to lack of full text in English or Spanish, and 20 because they addressed psychological programs preventing postpartum depression or maternal stress but did not focus on maternal role acquisition or transition to motherhood.

Finally, 11 studies met inclusion criteria, involving 1244 women and published between 2004 and 2022. Although no publication date restrictions were applied, earlier studies identified during the search were excluded because they did not meet the inclusion criteria, as they were primarily descriptive, lacked intervention designs, or did not assess maternal role acquisition or related outcomes.

**Figure 3:**
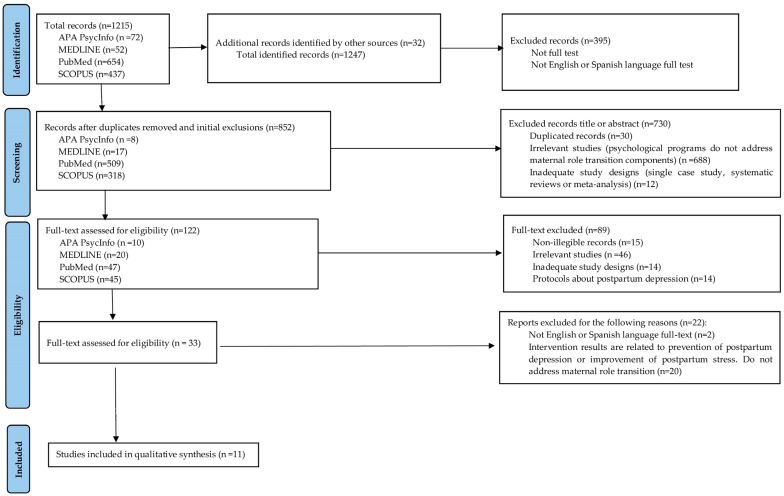
PRISMA flow diagram.

Five studies were conducted in Iran, three in China, one in Australia, one in Cyprus, and one in Spain. These studies evaluated psychological programs aimed at maternal role acquisition during pregnancy and postpartum. Table 1 and Figure 3 summarize the characteristics of the included studies.

Five studies employed an experimental design [19,20,21,24,25], while six utilized a quasi-experimental design [22,23,26,27,28,29]. All studies conducted their first evaluation in the third trimester of pregnancy and a second evaluation in the immediate postpartum period (2–4 weeks later). The program effects were primarily assessed using standardized instruments, except for one study that combined standardized tools with qualitative interviews [23]. Participants were adult women with higher education, and only two studies included the participation of their partners. Regarding obstetric characteristics, all participants were pregnant women at low obstetric risk; two studies involved women with unplanned pregnancies, seven studies included first-time mothers, and two studies focused on women with premature babies admitted to intensive care units.

The reporting of outcomes varied across studies, and standardized effect sizes and confidence intervals were not consistently available. Therefore, findings are presented descriptively based on the data reported in the original studies.

Standardized effect sizes and precision measures were not consistently reported across studies. Where sufficient data were available, effect sizes were calculated; otherwise, results are presented descriptively, and this limitation is explicitly acknowledged.

### 3.1. Effects on Maternal Role Acquisition

Five studies, with a total of 250 women, showed benefits in maternal role adjustment following their interventions. The programs consisted of 3 to 6 one-hour sessions, combining face-to-face sessions and telephone follow-ups 2 weeks, 4 weeks or 4 months postpartum. Interventions included group sessions, individual sessions and telephone follow-ups, ranging in duration from 30–45 min to 90 min modules. These sessions covered a variety of topics, such as childbirth preparation, stress management strategies, newborn care skills training and strengthening maternal self-efficacy. Only one study used booklets in support journals [23]. Most interventions focused on women, except the study by Hidalgo et al. [23] which included couples. Most sessions were group-based, except Heydarpour et al. [22] who conducted individual sessions. Kordi et al. [26] included an individual postpartum follow-up session, shorter than the prenatal sessions. Most programs started in the third trimester (week 33 onwards) and ended in the postpartum period, with individual telephone follow-ups.

Interventions focused on fostering maternal attachment through training in infant care behaviors and counseling on feeding and breastfeeding. In addition, self-knowledge about irrational beliefs about motherhood, emotional management, identification of realistic goals, valuing personal achievements and seeking parental support were promoted. Information was also provided on pregnancy, childbirth and puerperium, educational systems and vaccination schedules, strengthening confidence in the maternal role. Other aspects addressed included cognitive restructuring to modify dysfunctional thoughts, identification and readjustment of expectations about motherhood, and normalization of physical, emotional, marital and family changes, together with maternal role modeling and imagination techniques.

In relation to the evaluation of change in role acquisition, the Maternal Role Adaptation Scale was used and the self-report Self-Perception of Parental Role Scale. In addition, interviews were conducted at the beginning of pregnancy and during the first year of the baby’s life to collect information on dimensions relevant to the process of becoming a mother, such as knowledge and doubts, social support network, marital relationships, and satisfaction with parenthood. Fasanghari et al. [19] and Kordi et al. [26] assessed maternal role acquisition by summing the scores of three self-reports: the Myself as a Mother Scale, the My Baby Scale and the Perceived Competence Scale (PCS).

Across these studies, the programs were generally associated with improvements in maternal role acquisition measures, although not all changes reached statistical significance and standardized effect sizes were rarely reported. Fasanghari et al. [19] reported a statistically significant improvement in maternal role acquisition in the intervention group compared to the control group (*p* = 0.036). Heydarpour et al. [22] reported improvements in maternal self-efficacy following the intervention, although no standardized effect size was provided.

Hidalgo et al. [23] reported an increase in maternal competence. Özbiler & Beidoglu [28] found that the intervention improved subjective well-being and parental role perception. Sohrabi et al. [29] observed an improvement in maternal self-efficacy and a 12% reduction in postnatal depressive symptoms.

### 3.2. Effects on Maternal Competence and Maternal Self-Efficacy as Elements Related to the Maternal Role

Six studies with 994 women highlight maternal competence and self-efficacy as keys to adaptation to the maternal role. Maternal competence is measured with PCS, and self-efficacy, which reflects perceived parenting ability and emotional state, is assessed with the Maternal Self-Efficacy Questionnaire. These studies delivered their programs exclusively to women, except for one that offered optional participation by couples [24]. Four studies conducted group sessions [19,20,21,27] with individual and telephone follow-up. The study by Kavanagh et al. [25] used a self-guided telematics format. The programs began in the third trimester of pregnancy and ended postpartum, with between 3 and 6 sessions of approximately one hour each.

In terms of program content, training in caregiving and feeding skills were the most frequently addressed topics to foster maternal competence and self-efficacy. Interventions also addressed other issues such as communication and problem-solving patterns with the partner and soliciting social support for parenting. Along with these work points, others were added such as psychoeducation on childbirth and the emotional changes experienced at this stage. Although shared parenting was a recurring theme in the studies, only two articles delved into this aspect through role sharing, self-efficacy, maternal competence and social support. Kavanagh et al. [25] program focuses on increasing self-efficacy through social support and maintaining partner relationship satisfaction during the perinatal period. Mothers can access these resources online and at their own pace. On the other hand, Gao et al. [21] worked on perceived social support to foster maternal role competence, with the aim of improving maternal and spousal psychological well-being in the face of shared parenting demands.

Studies generally suggest improvements in maternal competence and self-efficacy, although not all findings have reached statistical significance. Gao et al. [21] observed improvements in maternal competence and reduction in depressive symptoms. Karami et al. [24] reported higher maternal self-efficacy scores in the intervention group compared to the control group. However, this difference did not reach statistical significance (35.6 ± 5.7 vs. 30.9 ± 1.9, *p* = 0.08) and child-care behavior (78.9 ± 3.8 vs. 76.2 ± 3.6, *p* < 0.001). Kavanagh et al. [25] reported increases in self-efficacy to provide support (*p* = 0.01; d = 0.26) and relationship satisfaction (*p* = 0.03; d = 0.20).

### 3.3. Effects of the Programs on Perinatal Mental Health

A positive side effect identified in six studies of maternal role support programs was a reduction in postpartum depressive symptoms. In all studies, the Edinburgh Postnatal Depression Scale was used to assess postpartum depressive symptomatology.

The studies showed significant improvements in postnatal depressive symptomatology compared to the control groups. Fasanghari et al. [19] reported a 24% reduction in depressive symptoms in the intervention group versus 10% in the control group (*p* = 0.036). Gao et al. [20] observed a 30% decrease in postnatal depression in the intervention group, compared to 12% in the control group (*p* < 0.05). Karami et al. [24] found a 12% reduction in depressive symptoms in the intervention group, compared to 5% in the control group (*p* < 0.001). Kavanagh et al. [25] reported improvements in self-efficacy and a reduction in relationship dissatisfaction in the intervention group (*p* = 0.01; d = 0.26), compared to smaller improvements in the control group (*p* = 0.03; d = 0.20). Gao et al. [21] also highlighted a significant decrease in postnatal depression in the intervention group compared to the control group (*p* < 0.05). Another significant health effect was found in perceived stress scores. The study by Heydarpour et al. [22] revealed a considerably significant reduction in perceived stress in mothers with preterm infants hospitalized in the neonatal intensive care unit. Stress scores on the Perceived Stress Scale decreased markedly in the intervention group compared to the control group (*p* < 0.05), in addition to a substantial improvement in maternal role adjustment.

### 3.4. Certainty of Evidence

The GRADE assessment indicated that the overall certainty of the evidence for both maternal role acquisition and maternal self-efficacy/competence outcomes ranged from low to very low. This was primarily due to the high risk of bias across several included studies, the heterogeneity of interventions and outcome measures, the predominance of non-randomized designs, and the lack of precision measures such as confidence intervals and standardized effect sizes.

## 4. Discussion

The systematic review examined the impact of psychological support programs for mothers, highlighting the benefits in promoting maternal role during pregnancy and postpartum, comparing measures taken in the third trimester, immediate postpartum (2 weeks postpartum) and late puerperium (4–6 months postpartum). These findings reinforce the relevance of addressing maternal role acquisition as a dynamic developmental process rather than as a static outcome measured exclusively in the postpartum period. It emphasized the importance of personalized professional support tailored to each woman’s unique needs. Effective interventions should consider the mother’s specific requirements, her psychobiography, values, fears, and the support needed from her close environment. This individualized approach is consistent with current models of person-centered perinatal care and has direct implications for clinical practice within maternal health services.

Review highlights the central role of women in psychological support programs, which have shown significant benefits in maternal role acquisition, as well as maternal competence and self-efficacy [19,22,24]. Maternal competence and self-efficacy appear to function as core mechanisms facilitating successful adaptation to the maternal role, rather than as secondary outcomes. In addition, it is crucial to mention infant well-being. Studies indicate an improvement in mother–baby bonding, where the baby receives attention, and the mother reinforces her perceived competence. This reciprocal relationship between maternal well-being and infant development underscores the bidirectional nature of maternal role acquisition. Since each mother and baby have unique needs, the participation of both in professional accompaniment is essential. Gao et al. [21] observed improvements in maternal competence at 6 months postpartum due to childbirth preparation and newborn care. Other studies, such as Hidalgo et al. [23], demonstrated improvements in mother–baby interaction after training sessions. Kavanagh et al. [25] reported increased satisfaction with mother–baby interaction at 3 and 6 months postpartum. Karami et al. [24] program, based on Bandura’s self-efficacy theory [30], focused on skills training, addressing newborn care, techniques to strengthen mother-infant interaction, strategies to manage stress and anxiety, and the development of practical infant care skills. These results support self-efficacy as a theoretically grounded and clinically relevant target for perinatal interventions.

Across the included studies, several common intervention components can be identified, including psychoeducation on motherhood and infant care, emotional support, enhancement of maternal self-efficacy, and strategies to improve social and partner support. Despite variations in delivery format (e.g., group versus individual, face-to-face versus remote) and program duration, these shared elements appear to underpin the observed improvements in maternal role acquisition and related outcomes.

However, Ngai et al. [27] found no significant improvements in maternal role acquisition or some of its components during follow-up measures. This variability in outcomes highlights the heterogeneity of intervention designs and suggests that maternal role acquisition may be influenced by contextual, cultural, and methodological factors. Preliminary studies on caregiving training programs emphasize the importance of attachment work in enhancing mother-infant interactions and maternal role acquisition. Educating mothers on self-care and infant care effectively addresses emotional issues related to motherhood [31,32]. The studies highlight the need to incorporate emotional components in psychological support programs from gestation through postpartum. Additionally, considering the woman’s context and developmental stage is crucial for fostering secure attachment and allowing her to adapt to motherhood at her own pace. These findings align with transdiagnostic approaches to perinatal mental health, in which emotional regulation and identity integration play a central role.

Motherhood begins in pregnancy, where a pressure-free environment that favors the development of the baby and the woman’s new identity is crucial. This process continues in parenting, fostering connection and respecting the needs of each dyad. The woman relearns to identify her values and make decisions based on them to build her maternal role [9,33]. Some studies have developed dynamics working on attachment and self-awareness, helping mothers to understand their emotions and strengths. Fasanghari et al. [19] designed a program based on Mercer [2,3] that highlights the development of maternal identity, health and relationship with the father, focusing on caregiving skills and maternal confidence. Kordi et al. [26] and Sohrabi et al. [29] also developed programs focused on caregiving skills, mother–child interactions, and stress management. These findings reinforce Mercer’s theoretical framework as a valuable guide for the design of perinatal psychological interventions. Although studies included women who did not plan their pregnancy, the results suggest that the benefits in maternal confidence and role acquisition may be generalizable to any woman on her motherhood. This strengthens the preventive potential of such programs beyond traditionally defined risk groups.

Other programs, based on interpersonal psychotherapy [20,21], focused on addressing conflicts in mothers’ relationships and helping them to properly use social support to better manage stressful situations. In seeking social support, Heydarpour et al. [22] developed an educational program aimed at maternal role adoption, providing emotional, informational, and practical support to reduce perceived stress. The results of these programs, along with other studies involving the participation of couples [23,25], highlight the importance of guiding fathers to provide a loving and supportive space for the mother, emphasizing how essential their parental role is in the transition to motherhood. From a health promotion perspective, incorporating partners into perinatal interventions may enhance program effectiveness and sustainability.

In this context, it is important to consider the role of culture in the design and implementation of these programs. The present review highlights the predominance of Eastern culture in the development of perinatal support programs focused on maternal role acquisition. Previous literature pointed out that intrapersonal and interpersonal relationships in perinatal health, such as family or conjugal relationships, are central values in Eastern culture. Therefore, professional work in accompaniment focusing on these aspects is considered appropriate in Eastern countries [34,35,36] and extensible to understanding the demands of mothers in Western countries [37,38]. Nonetheless, further culturally sensitive research is needed to adapt and validate these interventions across diverse healthcare systems and sociocultural contexts.

Therefore, the alignment of the results collected in this review suggests that professional accompaniment in introspection is key for an adequate transition to motherhood, also benefiting the prevention of perinatal and postnatal depressive symptoms [15]. Framing maternal role acquisition as a protective factor positions these programs as promising preventive strategies within perinatal mental health care. In addition, the presence of the partner is essential to meet the needs of the dyad. In this context, it is important to welcome the new emerging identity in him or her so that he or she can provide the necessary care for the new mother. Therefore, it is essential to facilitate group accompaniment spaces that integrate fatherhood as a support for motherhood. Such integrative approaches may contribute to improved maternal, infant, and family health outcomes at a population level.

### Strengths and Limitation

To our knowledge, this is the first review that compiles evidence on programs that encourage maternal role acquisition as a health promotion measure for women who decide to become mothers. By shifting the focus from symptom reduction to identity integration, this review contributes a novel perspective to perinatal mental health research. The search strategy made it possible to include quality studies that contemplate professional accompaniment from a humanistic attitude, with respectful and attentive listening to the needs of mothers. By focusing on the reconstruction of identity through the acquisition of the maternal role, motherhood is conceived as an experience of high personal and social impact. From this perspective, professionals are encouraged to approach motherhood from the perspective of individuality, where the role of the professional accompanier is to support the woman individually and empower her in the process of giving life and actively participating in the vital development of her baby. This approach is consistent with contemporary healthcare models emphasizing empowerment and shared decision making. At the methodological level, substantial heterogeneity, and all studies were peer-reviewed. Access to full text also allowed us to adequately review the designs and contents of the psychological support programs.

It should be noted that this study has some limitations. First, the review protocol was not prospectively registered, which may affect transparency and increase the risk of bias in the review process. However, predefined eligibility criteria and procedures were consistently applied throughout the study. Second, the number of included studies was limited. Third, 8 of the 11 studies focused on women from Eastern countries (Iran and China).

This cultural concentration should not be interpreted solely as a limitation in representativeness, but as a factor that may actively influence both the content of the interventions and the mechanisms through which they exert their effects. Constructs such as maternal identity, social support, and partner involvement are deeply embedded in cultural norms, family structures, and healthcare systems.

In collectivist cultural contexts, where interdependence and family cohesion are emphasized, interventions centered on relational adjustment, social support, and role expectations may be particularly effective. However, in more individualistic contexts, where autonomy and self-concept are more strongly emphasized, these constructs may operate differently, potentially affecting the relevance and impact of similar interventions.

Therefore, caution is warranted when interpreting and generalizing these findings beyond the cultural contexts in which the studies were conducted. While some components of these interventions may be transferable, their applicability to Western populations remains a hypothesis that requires further empirical validation. Future research should aim to replicate and adapt these interventions across diverse cultural and healthcare settings.

In addition, the variability in intervention formats and outcome measures precluded quantitative synthesis, underscoring the need for more standardized approaches in future research.

The substantial clinical and methodological heterogeneity across the included studies, including differences in intervention formats, outcome measures, and study designs, precluded the use of meta-analysis and limited the ability to quantitatively synthesize the findings.

The GRADE assessment further indicated that the certainty of the evidence was low to very low for the primary outcomes. This reflects the predominance of quasi-experimental designs, the high risk of bias in several studies, and limitations in reporting precision measures. These factors limit the strength of conclusions and highlight the need for more rigorous and standardized research in this field.

Another limitation concerns the study selection process. Although verification steps were implemented, the initial screening of titles and abstracts was conducted by a single reviewer rather than independently by two reviewers, as recommended in systematic review guidelines. This may increase the risk of selection bias or omission of relevant studies. Future reviews should incorporate independent dual screening at all stages to enhance methodological rigor. Future research should prioritize randomized controlled trials with active comparators, incorporate blinding of outcome assessment where feasible, and use standardized measures of maternal role acquisition. In addition, longer follow-up periods (e.g., ≥12 months) and the inclusion of diverse cultural and socioeconomic populations are needed. Economic evaluations could also be embedded within primary studies to assess cost-effectiveness.

Finally, another important limitation concerns the incomplete reporting of effect sizes and precision measures (e.g., confidence intervals) in the included studies. In many cases, insufficient statistical information prevented the calculation of standardized effect sizes, which limits the comparability and quantitative interpretation of the findings.

## 5. Conclusions

In summary, this systematic review highlights the importance of working on maternal role acquisition and its benefits on women’s mental health, especially in reducing postpartum depression and perceived stress. Maternal role acquisition emerges as a key protective factor in the perinatal period, with relevance not only for psychological well-being but also for maternal–infant interaction and broader family adjustment. Psychological support programs have been shown to be effective in improving maternal competence and self-efficacy, as well as in strengthening perceived social support. These variables appear to mediate the adaptive integration of motherhood, supporting women in managing the emotional, cognitive, and relational demands of this transition.

The included studies highlight the need for an individualized and culturally sensitive approach to maternal support. Evidence suggests that respectful psychological support can empower women in their transition to motherhood, improving both their well-being and that of their babies. From a healthcare perspective, integrating psychological accompaniment focused on maternal role acquisition into routine perinatal care may represent a feasible and promising preventive approach for promoting maternal and child health. However, further research is needed to confirm the generalizability of these results to different cultural and social contexts, which will allow the development of more inclusive and effective interventions. Future studies should prioritize longitudinal designs, standardized outcome measures, and the inclusion of partners and diverse family structures to strengthen the evidence base and guide the implementation of maternal-role-focused programs within healthcare systems.

## Figures and Tables

**Table 1:** Characteristics of included studies (n = 11).

Author	Country	Study Design	N (Intervention/Control)	Intervention Components	Comparator	Variables and Instruments	Effect Size	Attrition	Blinding
Fasanghari et al., 2018 [19]	Irán	Randomized controlled trial	34/33	Maternal role training based on Mercer’s theory (group + individual sessions, follow-up)	Routine care	Maternal identity (Myself as Mother Scale)	Insufficient data for calculation	NR	NR
Gao et al., 2012 [20]	China	Randomized controlled trial	97/97	Interpersonal psychotherapy-based childbirth education program	Standard care	Maternal competence (PSOC)	Insufficient data for calculation	NR	NR
Gao et al., 2015 [21]	China	Randomized controlled trial	97/97	Postnatal interpersonal psychotherapy-based intervention	Standard care	Maternal competence (PSOC)	Insufficient data for calculation	NR	NR
Heydarpour et al., 2022 [22]	Irán	Quasi-experimental study	30/30	Educational and supportive intervention (NICU context)	Usual care	Maternal role adaptation	Insufficient data for calculation	NR	NR
Hidalgo et al., 2004 [23]	España	Quasi-experimental study	Promote a successful transition to parenthood through a support and training program, and to assess its impact on parents’ satisfaction and the quality of interactions with their infants	Distance-based educational program (“Born to Life”)	Comparison group	Parent satisfaction/interaction quality	Insufficient data for calculation	NR	NR
Karami et al., 2020 [24]	Irán	Randomized controlled trial	34/34	Skills-based counseling sessions (self-efficacy training)	68 pregnant nulliparous or multiparous women.	Maternal self-efficacy (Questionnaire)	Insufficient data for calculation	NR	NR
Kavanagh et al., 2021 [25]	Australia	Randomized controlled trial	194/194	Online self-guided program (Baby Steps)	Information-based program	Self-efficacy/relationship satisfaction	d = 0.26; d = 0.20	NR	NR
Kordi et al., 2017 [26]	Irán	Quasi-experimental study	34/33	Maternal role training program (group + follow-up)	Routine care	Maternal role attainment (PCS)	Insufficient data for calculation	NR	NR
Ngai et al., 2009 [27]	China	Quasi-experimental study	42/41	Psychoeducation program based on resourcefulness	Routine care	Maternal competence (PSOC)	Insufficient data for calculation		NR
Özbiler and Beidoğlu, 2020 [28]	Chipre	Quasi-experimental study	30/30	Subjective well-being intervention	Control group	Parental role perception	Insufficient data for calculation		NR
Sohrabi et al., 2021 [29]	Irán	Quasi-experimental study	32/31	Training program (preterm infant context)	Routine care	Maternal role adaptation	Insufficient data for calculation		NR

## Data Availability

No new data were created or analyzed in this study. Data sharing is not applicable to this article.

## Supplementary Materials

The following supporting information can be downloaded at: https://www.mdpi.com/article/10.3390/healthcare14131958/s1, Table S1: PRISMA Checklist; Table S2: Full search strategies; Table S3: Risk-of-bias assessment; Table S4: GRADE Summary of Findings tables for key outcomes.
